# Reduction of resources with mRNA host-response whole blood testing on patients presenting to the emergency department with suspected infections: a retrospective analysis of 30 cases

**DOI:** 10.1099/jmm.0.002085

**Published:** 2025-10-17

**Authors:** Alan H.B. Wu, Shane Falcinelli, Rama Yakubu

**Affiliations:** 1 Department of Laboratory Medicine, University of California, San Francisco, CA, USA; 2 Core Laboratory, Zuckerberg San Francisco General Hospital, University of California, San Francisco, CA, USA; 3 Microbiology Laboratory, University of California, San Francisco, CA, USA

**Keywords:** bacterial and viral infection, host response, resource utilization, sepsis

## Abstract

**Introduction.** The TriVerity™ test (Inflammatix Inc.) uses the patient’s mRNA expression patterns from whole blood to provide a site-agnostic likelihood of bacterial infection and/or viral infection. The test also reports an illness severity score, an all-cause predictor of the need for critical care (mechanical ventilation, vasopressor therapy or renal replacement therapy) within 7 days of testing.

**Hypothesis/Gap Statement.** Differentiating the source and assessing disease severity in patients presenting to the emergency department (ED) with signs and symptoms of sepsis remains an unmet medical need.

**Aim.** The results of the TriVerity test were compared to the EPIC sepsis score, systemic inflammatory response syndrome criteria, discharge diagnoses, location of patient discharge and an assessment of potential cost savings in unnecessary resource utilization.

**Methodology.** Blood was retained from 30 patients presenting to the ED with signs and symptoms suggestive of sepsis. Remnant EDTA-preserved blood from routine medical testing was transferred into PAXgene tubes, stored frozen and retrospectively tested with TriVerity.

**Results.** TriVerity was correctly correlated with clinical diagnosis and outcomes in 29 of 30 patients with one false negative (low severity score for sepsis diagnosis, admitted for bladder cancer and on pre-existing antibiotic therapy). For two patients with a positive TriVerity bacterial and severity score, antibiotic treatment may have been initiated 6–9 h earlier if tested prospectively. Twelve patients were discharged home with a mean ED length of stay of 7.25 h, of which 7 (58 %) had a positive EPIC sepsis score (>5) and all had a low TriVerity severity score. If this test were performed prospectively at ED presentation, a net savings of $351 per patient is estimated in direct laboratory, radiology and ED costs alone, without consideration of potentially shorter hospital stays or changed disposition.

**Conclusion.** The TriVerity test may enable early consideration of ruling in or out of bacterial and/or viral infection, provide severity assessment of adult patients presenting to the ED suspected of, and help reduce unnecessary utilization of laboratory and ED resources.

## Introduction

Sepsis is a life-threatening organ dysfunction caused by a dysregulated host response to infection [1]. According to the World Health Organization, there are 50 million cases per year with over 11 million deaths [2]. In the USA, the average cost of treating a patient with sepsis was $28,800 in 2021 [3]. Patients often present to the emergency department (ED) with non-specific signs and symptoms of infection. The difficulty in discriminating between bacterial, viral and non-infectious states is a common problem. A national estimate of all ambulatory visits suggests that 30% of all antibiotics are given to patients without bacterial infections, underscoring the difficulty [4]. The systemic inflammatory response syndrome (SIRS) criteria were established in 1992 and include the presence of fever, abnormal white cell count, a low carbon dioxide [partial pressure of carbon dioxide (PCO_2_)], rapid heart rate and rapid respiratory rate [5]. Due to the low specificity of the criteria for sepsis, other classification tools have been developed, including the Sequential Organ Failure Assessment (SOFA) and the quick SOFA (q-SOFA) score as part of the Sepsis-3 definition [6]. However, none of these scores have the requisite performance to reliably determine which patients will progress to sepsis and require intensive care unit (ICU) admission, making risk stratification and disposition decisions an ongoing challenge for ED physicians [7].

The gold standard for assessment of bacterial bloodstream infection is blood cultures [8]. However, these cultures are prone to false positives due to contamination, and results are not usually available within the first 24 h of ED presentation, a critical time in patient management. Additionally, sepsis can be associated with organisms that do not grow in standard blood cultures, and with non-bloodstream infections such as a bacterial pnemonia [9].

For hospitals using the Epic Electronic Medical Record System (Verona, WI), the Sepsis Score has been made available to assist caregivers. It uses a proprietary algorithm to predict sepsis risk. This score was developed from 400,000 patient encounters incorporating 80 demographic, vital signs and standard laboratory tests. The algorithm used is proprietary; therefore, a detailed description is not available [10,11]. The performance of this algorithm, however, has been variable. For instance, Cull *et al*. evaluated 11,512 patients and found a sensitivity, specificity, positive predictive value (PPV) and negative predictive value (NPV) of 86%, 81%, 34% and 98 %, respectively, and an area under the receiver operating characteristic (AUC-ROC) curve of 0.83 using a sepsis score cutoff of 5.0 [10]. In contrast, using a sepsis score cutoff of 6.0, Wong *et al*.’s evaluation of 27,697 patients demonstrated sensitivity, specificity, PPV and NPV values of 33%, 83%, 12% and 95%, respectively, with an AUC-ROC of 0.63 [11]. While the EPIC sepsis score is readily available to users of this electronic medical record system, its sensitivity and specificity vary significantly with small changes in cutoff values. This variability makes the tool difficult to apply in clinical practice without a high risk of error. Therefore, additional tools are needed for evaluation of sepsis in patients presenting to the ED. To increase the clinical sensitivity, we used a sepsis score of 5.0 as the cutoff for this study.

Several new commercial diagnostics leverage the host immune response to improve early diagnosis or prognosis of patients with suspected infection or sepsis. Food and Drug Administration (FDA)-cleared options include MeMed BV, a three-protein test that produces a ‘bacterial vs. viral’ score [12], the monocyte distribution width [13] and the IntelliSep test [14], each of which measure white blood cell physical properties to produce a risk of ‘sepsis,’ and SeptiCyte Rapid, a two-mRNA test which is intended to identify sepsis vs. non-infectious SIRS in ICU patients [15]. Here, we studied the TriVerity test (Inflammatix, Inc., Sunnyvale, CA), which measures 29 mRNA markers reflecting early innate immune system response and produces three numeric scores for the likelihood of a bacterial infection, the likelihood of viral infection and the likelihood of patient progressing to severe illness, defined as the all-cause need for mechanical ventilation, vasopressors or renal replacement therapy within 7 days [16]. The three TriVerity and range from 0 to 50 and is split into 5 ‘interpretation bands,’ from very low (0–10), low (11–20), moderate (21–30), high (31–40) and very high (41–50). Scores in the very low and low bands indicate a lower likelihood of infection or decompensation, respectively, whereas scores in the high and very high bands indicate a higher likelihood of infection or decompensation.

In this study, we evaluated 30 patients who presented to the ED with suspected sepsis and assessed TriVerity results in relation to their infection history, clinical laboratory testing and final disposition.

## Methods

### Subjects

Thirty adult subjects presenting to the Zuckerberg San Francisco General Hospital ED from 3 January to 6 February 2025, with suspected sepsis, were included based on the EPIC sepsis score and SIRS criteria. There were 19 males (mean age 52±17 years) and 11 females (mean age 61±19 y). At the time of ED presentation, and as part of routine medical care, 4 ml of blood was collected in EDTA tubes for a complete blood count (CBC) testing. After CBC testing, and within 1 h of blood collection, 2.5 ml of the remaining blood was aliquoted into a PAXgene tube (Becton Dickinson, Franklin Lakes, NJ) to preserve mRNA and then frozen at −20 °C. De-identified samples were sent to Inflammatix Inc. for TriVerity testing who provided test results without a charge. They were blinded to any clinical findings and laboratory results.

### Clinical adjudication

A medical record review was performed for each patient by the authors of this study. The patient’s vital readings were retrieved from the patient’s electronic medical records (Epic, Verona, WI) and include blood pressure, temperature, heart rate and respiration rate. Blood was collected within 1–2 h after presentation, and the results using the hospital’s standard of care assays were obtained for the white blood count, venous PCO_2_, lactate, procalcitonin, high-sensitivity C-reactive protein, B-type natriuretic peptide, high-sensitivity cardiac troponin I and results for blood cultures, influenza A and B and coronavirus disease 2019 (COVID-19) nasal. The EPIC sepsis score was recorded before, during and after the initial blood collection and at approximately 1 hour thereafter. Following the patient’s ED and hospital discharge, the number of times each patient was readmitted to the ED was determined over the ensuing 1 and 6 months.

### Statistics

A student’s t-test was used using MedCalc ver 19.6.4 (Ostend, Belgium) for proportions and means. A *P*<0.05 was considered significant.

## Results

Table 1 summarizes the demographics, presence of SIRS criteria, EPIC Severity scores and TriVerity test scores. The data are divided by the location of each patient’s ED discharge (home, or the combination of hospital ward or ICU). The latter category was combined because of the small number of ICU admissions (*n*=2). There was no difference between these groups in the age, gender, the overall number of abnormal SIRS criteria present, and the individual incidence of abnormal SIRS parameters: increased heart rate (>90 beats/min), abnormal temperature (<36 or >38 °C), abnormal white blood cell count (<4.0 or >12×10^9^/l) and either increased respiration rate (>20 breaths/min) or low PCO_2_ (<32 mmHg). Although not statistically significant, the EPIC sepsis score for the overall group increased from admission (pre-blood collection) to the time of the initial blood collection and the two subsequent readings (taken within an hour hereafter). The home discharge patient score decreased over time, while the ward/ICU discharge patients’ score increased over time (*P*=ns). The TriVerity bacterial score was statistically lower for the home discharge group than the admitted group (*P*<0.001), while the viral score was statistically higher for the home discharge group. The severity score was higher in the admitted group (12.2 vs. 20.1, Table 1), but the results did not reach statistical significance.

**Table 1.** Demographics of patients tested

Parameter	Overall	Home discharge	Ward/ICU discharge	*P* value*
No. of subjects	30	14	16	ns
Age, years (±sd)	56±18	56±20	55±18	ns
Gender (males, %)	19/30 (63%)	9/14 (64%)	10/16 (62%)	ns
Abnormal temperature <36 or >>38 °C, (%)†	18/30 (40%)	6/14 (43%)	6/16 (37%)	ns
Heart rate >90 beats/min	28/30 (93%)	13/14 (93%)	15/16 (94%)	ns
Respiration rate >20 breaths/min	18/30 (56%)	10/14 (71%)	8/16 (50%)	ns
White blood count count <4 or >12×109/l	17/30 (57%)	6/14 (43%)	11/16 (69%)	ns
PCO2 <32 mmHg	4/30 (13%)	1/14 (7%)	3/16 (19%)	ns
Number of SIRS criteria present (sd)	2.57±0.60	2.50±0.52	2.71±0.83	ns
Epic Score (sd)				
Admission	4.80±3.42	3.89±2.20	5.67±4.36	ns
Initial blood draw	5.24±2.65	4.89±1.66	5.65±3.53	ns
Subsequent #1++	5.47±2.74	4.69±1.76	6.38±3.44	ns
Subsequent #2	5.59±3.56	4.07±2.39	6.94±4.21	ns
TriVerity score (sd)				
Bacterial	22.8±14.0	15.811.0	29.4±12.7	*P*<0.001
Viral	20.5±15.4	27.4±17.6	13.0±6.8	*P*=0.005
Severity	16.7±11.3	12.2±7.8	20.1±14.5	ns

*Home vs. Ward/ICU discharge.

†One patient was hypothermic, the remainder were hyperthermic.++The Subsequent Epic scores refer to values obtained at 1 and 2 h after the score taken at the time of the CBC blood collection.

Table 2 lists the EPIC sepsis scores and TriVerity scores for each patient. Of the 30 patients studied, there was only 1 case where the TriVerity result was inconsistent with the history (patient #19), producing an overall accuracy of 96.6% (29/30). There were two cases (#4 and #16) where the bacterial and viral score was a mismatch with lab tests but correctly reflected the actual hospital course. Patient #4 was positive for influenza A with a low viral score and a high bacterial score and was appropriately treated with intravenous antibiotics for 4 days. Patient #16 showed a high bacterial score with no source of infection identified and no prior antibiotic treatment. However, both patients had an intermediate severity score and stayed for 5 and 7 days, respectively, reflecting the severity of their admission. For the remaining 27 cases, TriVerity correlated with the discharge medical record (7 rule out, 12 bacterial and 8 viral). 

**Table 2.** Comparison of EPIC sepsis score with TriVerity

Case no.	EPIC score*				TriVerity†				
	1^st^	2^nd^	3^rd^	4^th^	Bact.	Viral	Severity	Disposition	Discharge diagnosis
1	**8.84**	**7.72**	**8.81**	**10.04**	43	5	8	Ward	Sepsis, pneumonia
2	1.50	2.95	1.76	1.76	8	18	7	Ward	Heart failure
3	3.79	3.79	2.13	3.16	14	15	8	Ward	Heart failure, atrial fib‡
4	3.13	3.13	1.83	1.52	38	11	27	Ward	Influenza A, ketoacidosis
**5**	**6.91**	**6.91**	**5.59**	**5.68**	8	44	10	ICU	Influenza A, heart failure
6	2.88	2.88	2.16	0.97	13	12	10	Home	Diabetes, lactic acidosis
7	3.39	**5.37**	**5.44**	3.39	26	9	14	Home	Peritonsillar abscess
8	1.84	4.58	4.30	4.30	39	11	28	Home	Cellulitis
9	**2.29**	**2.29**	**13.6**	**13.86**	30	15	15	Ward	Urinary tract infection
10	2.47	2.47	2.31	0.69	18	9	6	Home	Cellulitis
11	3.28	3.05	3.37	**7.80**	45	5	38	ICU	Septic shock
12	**5.10**	**5.10**	4.40	4.40	11	10	5	Home	Chronic pancreatitis
13	4.10	**8.20**	**8.20**	**8.20**	40	6	31	Ward	Pneumonia, chronic obstructive pulmonary disease
14	4.53	4.38	4.48	3.47	7	46	11	Home	COPD
15	**6.05**	**5.86**	**5.86**	**12.38**	43	14	38	Ward	Severe sepsis
16	3.15	3.22	**7.92**	**7.95**	36	5	29	Ward	Heart failure, pancreatitis
17	**11.50**	**14.97**	**9.89**	**12.52**	43	4	30	Ward	Sepsis, cellulitis
18	**8.69**	**8.29**	**8.36**	**10.27**	4	47	5	Home	COPD
19	4.12	4.30	**6.23**	**6.23**	20	24	6	Ward	Septic shock, pyelonephritis
20	2.66	2.66	2.72	2.72	25	22	16	Ward	Scalp carbuncle debridement
21	0.94	**6.33**	**6.33**	**5.95**	10	45	13	Home	COPD, pneumonia
22	4.74	4.32	4.06	4.06	5	47	6	Home	Influenza A
23	1.53	2.58	2.54	**5.29**	36	10	29	Home	COPD
24	**7.49**	**7.26**	**7.42**	**6.63**	41	22	43	Ward	Influenza A, HIV
25	3.84	5.65	5.65	3.22	9	39	5	Home	Influenza A
26	**7.11**	**7.11**	**6.89**	4.17	18	14	13	Home	Nausea, vomiting
27	2.17	4.31	4.04	**5.31**	7	46	11	Home	Influenza A
28	5.29	**5.03**	4.73	1.48	18	39	15	Home	Influenza A
29	**17.31**	**7.63**	**7.63**	**7.79**	37	9	12	Ward	COPD
30	3.38	**5.37**	**5.37**	2.45	18	12	12	Ward	Colon cancer

*1st: score before the collection of the CBC, 2nd score at the time of the CBC, 3rd and 4th are taken about 1 h after, respectively. Results exceeding 5.0 are bolded.

†Triverity score. Intermediate 20–29 (blue), high 30-50 (red).

‡Afib, atrial fibrillation; COPD, chronic obstructive pulmonary disease; HIV, human immnodeficiency virus. The ED length of stay for patients #6, 7, 10, 12, 18, 21, 26, and 28 were 9, 21, 28, 4, 6, 9, 23, and 9 h, respectively.

The case where the TriVerity result was inconsistent with the clinical history involved a 35-year-old male with bladder cancer (T4N3M0, Tanner Stage 4, patient #19 from Table 2) and a history of *Proteus mirabilis* urinary tract infections (UTIs). A few weeks before his presentation, he was treated with gemcitabine. Two days prior to presentation to the ED, he reported subjective fevers, chills and burning with urination. Vital signs showed a fever of 38.1 °C, heart rate of 128 beats/min, blood pressure of 106/68 mmHg and PCO_2_ of 44 mmHg. His lactate and white blood cells were normal at 1.0 mmol l^−1^ and 4.8×10^9^/l, respectively, and his initial EPIC sepsis score was <5.0. He was treated with ertapenem at the time of blood culture collection and 2 h after his blood was collected for TriVerity testing. He required 1 day in the ICU prior to transfer to a hospital ward and was discharged on hospital day 6. His TriVerity scores were bacterial 20, viral 24 and severity 6. It is possible that the patient’s prior infusion with a chemotherapeutic agent may have altered his immune function as described with gemcitabine [17] since lactate, EPIC sepsis score and TriVerity were all falsely negative. In other studies, the TriVerity SEPSIS-SHIELD study showed no general drop in performance with active chemotherapy [18]. In addition, this discrepant result may have been impacted by the initiation of partially effective antibiotic therapy before ED presentation and was put on a trial of methenamine, a non-antibiotic medication used to suppress recurrent UTI. A single false negative severity score was also described in an early pilot study of the TriVerity test, suggesting a relatively rare event, consistent with the FDA test labelling [19]. The test is meant to be used with clinical judgement, not as the sole tool to determine treatment.

Table 3 lists the tests and procedures that could potentially have been avoided if blood collected at the time of the patient’s ED presentation had been tested with TriVerity, assuming release of the TriVerity score within 1 h after collection (sample run time is ~30 min). The use of the TriVerity score may help reduce unnecessary testing. For instance, test scores that were indicative of bacterial infection without concurrent viral infection (cases #1, 7–9, 11, 13, 15–17, 23 and 29) could have obviated the need to conduct viral testing. Similarly, in patients presenting with shortness of breath and a high viral score, without additional features concerning for bacteraemia, TriVerity may help reduce unnecessary blood culture testing. Furthermore, if there are no additional clinical features suggestive of acute coronary syndrome in a patient with shortness of breath and a clear bacterial or viral cause of illness based on TriVerity score, unnecessary serial cardiac troponin testing may be reduced. Using the 2025 Clinical Diagnostics Laboratory Fee Schedule, the total clinical laboratory savings was $4681 (Table 3).

**Table 3.** Cases showing possible tests and procedures ruled out by prospective use of TriVerity score

*Unnecessary lab test*		
Test/procedure	CPT code	Reimbursement
Cardiac troponin (patients #7, 10, 14, 21, 26, 28), 14 tests	84484	$12.47×14=$174.58
B-natriuretic peptide (patients #10, 21, 28), 5 tests	83880	$39.26×5=$196.30
Blood cultures x2 (patients #10, 26), 4 tests	87076, 87077	$10.32×4=$41.28
Urine culture (patients #6, 22), 2 tests	87086	$8.07×2=$16.14
Other culture (patients #7, #10), 3 tests	87075	$9.47×3=$28.41
Influenza A and B (patients #1, 6, 7, 9, 16, 17, 21, 23, 28, 29), 18 tests	87501	$153.93×18=$2,770.74
COVID-19 (patients #1, 7, 9, 10, 11, 13, 16, 21, 28, 29), 10 tests	87811	$41.38×10=$413.80
Novovirus (patient #15), 1 test	87798	$35.09
Rotavirus (patient #15), 1 test	87425	$11.98
Respiratory syncytial virus (patients #11, 23, 29), 3 tests	87634	$70.20x3=$210.60
Cytomegalovirus, IgG (patient #15), 1 test	86644	$14.39
Cytomegalovirus, IgM (patient #15), 1 test	86645	$16.85
*H. pylori* antibody (patient #26), 1 test	86677	$16.85
*H. pylori* stool antigen (patient #26), 1 test	87338	$14.38
Human immunodeficiency virus antigen (patient #26), 1 test	87389	$24.08
HIV antibody (patient #26), 1 test	86703	$13.71
Hepatitis panel (patient #26), 1 test	80074	$47.63
Hepatitis C (patient #10), 1 test	86803	$14.27
Syphilis antibody (patient #10), 1 test	86592	$4.27
Group A strep (patient #7), 1 test	87430	$16.81
Venous blood gas (patients #3, 7, 21, 26, 28), 8 tests	83807	$26.07x8 $208.56
Basic metabolic panel (patients #6, 7, 26, 28), 10 tests	80048	$8.46x10=$84.60
Lactate (patients #6, 7, 21, 26, 28), 7 tests	83605	$11.57x7=$80.99
Magnesium (patients #6, 26), 2 tests	83735	$6.70x2=$13.40
Phosphorus (patient #26), 1 test	84100	$4.47
Glucose (patients #6, 26, 28), 4 tests	82948	$5.04x4=$20.16
Creatine kinase (patient #10), 1 test	82550	$6.51
C-reactive protein (patient #10), 1 test	86140	$5.18
Erythrocyte sedimentation rate (patient #10), 1 test	85672	$2.70
CBC (patients #10, 21, 26, 28), 4 tests	85025	$7.77x4=$31.08
Acetaminophen (patient #26), 1 test	87389	$18.64
Ethanol (patient #26), 1 test	82077	$17.27
Toxicology screen (patient #10), 1 test	80305	$12.60
Toxicology confirmation (patient #10), 1 test	G0659	$62.14
Urinalysis (patients #10, 22, 26), 3 tests	81001	$3.71x3=$11.13
Alanine aminotransferase (patient #10), 1 test	84450	$5.18
Aspartate aminotransferase (patient #10), 1 test	84460	$5.30
Alkaline phosphatase (patient #10), 1 test	84075	$5.18
Bilirubin (patient #10), 1 test	82247	$5.02
Prothrombin time (patient #26), 1 test	85610	$4.29
**Subtotal, labs**		**$4681**
*Unnecessary procedures*		
CT pulmonary angiogram (patients #1, 13, 22, 27	71275	$262.97x4=$2984.40
Bedside ultrasound (patient #30)	76604	$56.92 x 2= $113.18
**Subtotal, procedures**		**$3,098**
**LOS 12 pt×$750×6/24 h (plus inflation adjustment from 2021)**		**$2,760**
**Total**		**$10539**
**Total per patient**		**$351**

Laboratory rates from 2025 Clinical Diagnostics Laboratory Fee Schedule, CLFS 2025 Q1V1. Radiology rates from 2024 Radiology rates from https://edge.sitecorecloud.io/americancoldf5f-acrorgf92a-productioncb02-3650/media/ACR/Legacy/864D0AA1B_032724_Impact-Tables-70000-codes_Marchupdate.pdf

CT, computed tomography; RSV, respiratory syncytial virus; SOB, short of breath.

With reference to unnecessary procedures, TriVerity testing with high viral scores (with very low or low illness severity and bacterial scores) may have the potential to sufficiently lower pre-test probability for pulmonary embolism such that the need for procedures such as CT pulmonary angiograms (×4) and bedside ultrasounds could have been reduced. The total savings was $3,098 (Table 3).

In 12 cases, a decision to discharge the patient might have been considered earlier if the presence of a very low or low TriVerity illness severity score had been known, for instance, by avoiding IV antibiotics and switching to PO (patients #6, 7, 10, 12, 14, 18, 21, 22, 25–28). The average length of stay (LOS) for these 12 patients was 7.25 h, with one patient (#10) having to stay overnight. The Agency for Healthcare Research and Quality has estimated the cost of $750 per day for an ED stay in 2021 [20]. To estimate potential savings, there was an additional 6 h added to the LOS, average of 6 h added to the LOS, accounting for 30 min to perform the test and 45 min to make a discharge decision. Applying this 6 h adjustment to each of the patients corresponds to a savings of $2760, when adjusted for inflation [21] (Table 3).

Other potential sources of cost savings include reducing unnecessary sepsis alerts (and the associated work and coding), shortening hospital LOS and avoiding unnecessary admissions. Four patients had ‘sepsis’ as part of the final diagnosis (#1, #15, #17 and #19), but all were admitted to a ward and not to the ICU. The LOS for these patients was 6 weeks, 4 days, 2 weeks and 6 days, respectively. Three of these patients (#1, #15 and #17) had very high TriVerity bacterial scores together with moderate-high TriVerity illness severity scores. The EPIC sepsis score was initially low (<5.0) for patients #11 and #19.

Further, nine patients had an EPIC score >5.0, triggering a sepsis alert, but with neither a charted diagnosis of sepsis nor requiring ICU admission. In these cases, the TriVerity test may have helped rule out sepsis with either a low bacterial score, low severity score or both. Turning off a ‘false positive’ sepsis alarm, with appropriate documentation, can save substantial resources in terms of nursing time and laboratory costs. The use of TriVerity may also help ensure appropriate Sepsis Measure 1: Severe Sepsis and Septic Shock – Early Management Bundle (SEP-1) compliance in the Centers for Medicare and Medicaid’s Value Based Purchasing programme. In this initiative, reimbursements are linked to SEP-1 performance. The financial impact is not modelled here [22].

There were two cases where an EPIC Sepsis Alert was falsely negative (<5.0), and the patients (#11 and 19) progressed to sepsis. There were two other cases where patients with severe bacterial infections did not receive antibiotics for several hours, but the TriVerity test would have indicated a bacterial infection. It is possible that earlier identification of these patients could have led to timelier treatment, potentially improving outcomes and shortening hospital LOS.

Combining savings from unnecessary laboratory tests, imaging and excess ED LOS, a total estimated cost avoidance was $10,539 averaging $351 per patient across all 30 subjects. This estimate does not account for potential additional savings related to reduced hospital days (as a result of reduced unnecessary therapeutics and/or treatment delays), or improved SEP-1 compliance.

Table 4 shows the correlation of initial ED disposition (home or hospital ward) to an ED return visit at 1 and 6 months. There was no statistical difference in incidence of an ED readmission with initial ED disposition with presence of a high SIRS score (≥3 criteria), or high EPIC risk score at the time of blood draw (>5.0). Only the abnormal TriVerity Severity score ≥30 was significant (14% vs. 56.0%, *P*=0.040). When the return visit rate was combined with any of these three scores, again, only the severity score was significant at 1 month (43% vs. 81%, *P*=0.032) and 6 months (43% vs. 88%, *P*=0.010).

**Table 4.** Rate of ED discharge location vs. an ED return visit (within 30 days and 6 months of discharge), presence of a high TriVerity severity score or either

	ED disposition		
Criteria	Home	Ward/ICU	*P* value
Univariate			
ED return visit (~1 month)	4/14 (29%)	4/16 (25%)	ns
ED return visit (within 6 months)	4/14 (29%)	10/16 (62.5%)	ns
SIRS criteria (≥3)	7/14 (50%)	7/16 (44%)	ns
High EPIC risk Score (≥5.0)	7/14 (50%)	9/16 (56%)	ns
Moderate/high TriVerity severity score (≥30)	2/14 (14%)	8/16 (56%)	0.040
Multivariate			
Either ED return 1 month or high SIRS	4/14 (29%)	12/16 (75%)	ns
Either ED return 6 months or high SIRS	9/14 (64%)	12/16 (75%)	ns
Either ED return 1 month or high EPIC score	7/14 (50%)	13/16 (81%)	ns
Either ED return 6 months or high EPIC score	7/14 (50%)	13/16 (81%)	ns
Either ED return 1 month or mod/high severity score	6/14 (43%)	13/16 (81%)	0.032
Either ED return 6 months or mod/high severity score	6/14 (43%)	14/16 (88%)	0.010

## Discussion

There exists no single criteria that can define sepsis or guide medical decisions. Recently, Al-Sultani *et al*. described the potential value of an electronic phenotype for sepsis, to include digital data from an electronic medical record, serum biomarkers and machine language algorithms to improve sepsis definition, early detection, prognostication and personalized treatment [23]. These authors discussed the need for data-driven insights rather than subjective descriptions and opinions.

A recent modified Delphi study on rapid sepsis identification in the ED concluded that a rapid sepsis test should provide an indication of the severity of dysregulated host immune response, assist in the risk stratification of patients presenting to the ED and be helpful in guiding initial clinical care, even if it does not identify the specific causative agent(s) [24]. As such, there have been several novel laboratory tests targeted to this area that have been developed and validated through clinical trials among patients who present to an ED with a suspicion of sepsis [12,16].

In this limited retrospective study, we highlight the potential utility of one commercial host response test in the potential triage of patients presenting to the ED with signs and symptoms of sepsis across domains of clinical decision-making, operational cost reductions and LOS. One surrogate for disease severity is the need to admit a potentially septic patient for further care. Another may be the frequency by which a patient returns to the ED due to continuing medical issues. For example, Chen *et al*. determined the medical factors of patients who initially presented to an ED with an infection (pneumonia, UTI and cellulitis) and who were re-admitted with a diagnosis of sepsis within 7 days of discharge [25]. There was no inclusion of any of the novel host-response biomarkers. In this study, we showed that the TriVerity severity score better correlated to discharge decisions and re-admission rate than either the EPIC risk score or fulfilment of the SIRS criteria (Table 4). While the generation of these latter scores is based on established vital signs and routine clinical laboratory tests (e.g. CBC and PCO_2_ level), our results show that they are not useful to predict disease severity or need to admit patients for continuing care.

Implementation of any new laboratory tests requires an economic justification. This study demonstrated a potential cost savings associated with use of the TriVerity test. The focus here was on direct ED costs, not considering potential hospital disposition changes. A prior health economic model comparing TriVerity with standard of care including procalcitonin suggested a per-test cost savings of US$1,974 per patient, which assumed roughly $300 in direct laboratory/ED savings and roughly $1,700 in reduced hospital days/reduced admissions [26]. Thus, the present study appears to support the prior economic model, without consideration of disposition. In the prior model, the average hospital LOS was 2.1 days (reduced to 1.4 days with TriVerity), compared to an average 4.5 days LOS in the present study, in which two-thirds of patients were admitted. This population thus appears to have a higher degree of severity than the modelled population. Neither study estimated the economic benefit of reduced false-positive ‘sepsis alarms’ and concomitant SEP-1 compliance gains.

Our study showed one false negative result (patient #19) presumably due to the presence of an immunosuppressed state. Immunosuppression appears to have variable effects on host response tests. TriVerity’s FDA clearance specifically examined immunocompromise and found no overall difference. The same is generally true of SeptiCyte, the test based on mRNA expression test [27]. The MeMed BV score includes novel biomarkers including tissue necrosis factor-related apoptosis-inducing ligand (TRAIL) and IFN gamma-inducible protein-10 (IP-10). As both of these proteins have a role in suppressing apoptosis and angiogenesis, respectively, and are dysregulated in cancer [28,29], this case may have interfered with the MeMed BV test.

Limitations of this study include small sample size, single centre, non-random patient selection and retrospective data analysis. The estimated cost savings are theoretical and are contingent on physician acceptance of the TriVerity results and willingness to act on them (e.g. administer antibiotics if the bacterial scores are high or discharge to home if the severity scores are low). Additionally, while clinical factors and diagnoses play an essential role in the decision to discharge, there are many non-clinical issues such as patient (e.g. social factors, health access and family preferences), physician (risk management, professional background, etc.) and system factors (e.g. protocols, communication and resources) that also play a role [30]. Nevertheless, if sepsis risk can be excluded quickly, process improvements may be developed to streamline patient discharge.

Future studies are underway evaluating clinical outcomes with interventional use of TriVerity (e.g. NCT06637904, ‘TIMED’ Study). Extending and confirming these patient and operational benefits will be key towards eventual protocolization on a broader scale.

## Abbreviations

AUC-ROC: area under the receiver operating characteristic
CBC: complete blood count
COVID-19: coronavirus disease 2019
ED: emergency department
FDA: Food and Drug Administration
ICU: intensive care unit
LOS: length of stay
NPV: negative predictive value
PCO_2_: partial pressure of carbon dioxide
PPV: positive predictive value
SEP-1: Severe Sepsis and Septic Shock – Early Management Bundle
SIRS: systemic inflammatory response syndrome
SOFA: Sequential Organ Failure Assessment
UTI: urinary tract infection

## References

[1] Singer M, Deutschman CS, Seymour CW, Shankar-Hari M, Annane D (2016). The third international consensus definitions for sepsis and septic shock (Sepsis-3). JAMA.

[2] World Health Organization (2024). Sepsis. https://www.who.int/news-room/fact-sheets/detail/sepsis.

[3] Owens PL, Miller MA, Barrett ML, Hensche M (2024). Overview of outcomes for inpatient stays involving sepsis, 2016-2021. Statistical Brief #306. https://hcup-us.ahrq.gov/reports/statbriefs/sb306-overview-sepsis-2016-2021.pdf.

[4] Fleming-Dutra KE, Hersh AL, Shapiro DJ, Bartoces M, Enns EA (2016). Prevalence of inappropriate antibiotic prescriptions among US ambulatory care visits, 2010-2011. JAMA.

[5] Bone RC, Balk RA, Cerra FB, Dellinger RP, Fein AM (1992). Definitions for sepsis and organ failure and guidelines for the use of innovative therapies in sepsis. *Chest*.

[6] Koch C, Edinger F, Fischer T, Brenck F, Hecker A (2020). Comparison of qSOFA score, SOFA score, and SIRS criteria for the prediction of infection and mortality among surgical intermediate and intensive care patients. World J Emerg Surg.

[7] Guido Marcello M, Tumolo MR, De Donno A, Verri T, Serio F (2016). In vitro diagnosis of sepsis: a review. Path Lab Med Int.

[8] Phua J, Ngerng W, See K, Tay C, Kiong T (2013). Characteristics and outcomes of culture-negative versus culture-positive severe sepsis. Crit Care.

[9] Singer M, Deutschman CS, Seymour CW, Shankar-Hari M, Annane D (2016). The third international consensus definitions for sepsis and septic shock (Sepsis-3). JAMA.

[10] Cull J, Brevetta R, Gerac J, Kothari S, Blackhurst D (2023). Epic sepsis model inpatient predictive analytic tool: a validation study. Crit Care Explor.

[11] Wong A, Otles E, Donnelly JP, Krumm A, McCullough J (2021). External validation of a widely implemented proprietary sepsis prediction model in hospitalized patients. JAMA Intern Med.

[12] Carroll KC (2024). Assessment of MeMed BV assays for differentiating between bacterial and viral respiratory infections. Expert Rev Mol Diagn.

[13] Kralovcova M, Müller J, Hajsmanova Z, Sigutova P, Bultasova L (2024). Understanding the value of monocyte distribution width (MDW) in acutely ill medical patients presenting to the emergency department: a prospective single center evaluation. Sci Rep.

[14] O’Neal HR, Sheybani R, Kraus CK, Self WH, Shah AM (2024). Cellular host response sepsis test for risk stratification of patients in the emergency department: a pooled analysis. Acad Emerg Med.

[15] Balk R, Esper AM, Martin GS, Miller RR, Lopansri BK (2024). Validation of SeptiCyte RAPID to discriminate sepsis from non-infectious systemic inflammation. J Clin Med.

[16] Whitfield NN, Hogan CA, Chenoweth J, Hansen J, Hsu EB (2024). A standardized protocol using clinical adjudication to define true infection status in patients presenting to the emergency department with suspected infections and/or sepsis. Diagn Microbiol Infect Dis.

[17] Larson AC, Doty KR, Solheim JC (2024). The double life of a chemotherapy drug: immunomodulatory functions of gemcitabine in cancer. *Cancer Medicine*.

[18] Liesenfeld O, Arora S, Aufderheide T, Clements C, DeVos E (2024). Rapid and accurate diagnosis and prognosis of acute infections and sepsis from whole blood using host response mRNA amplification and result interpretation by machine-learning classifiers. Nature Port.

[19] Lu J, ter Voert MA, Ünal M, Whitfield NN, Liesenfeld O (2025). Early sepsis recognition: a pilot study using a rapid high-multiplex host-response mRNA diagnostic test. ICMx.

[20] Roemer M (2024). Costs of treat-and-release emergency department visits in the united states, 2021, statistical brief #311. https://hcup-us.ahrq.gov/reports/statbriefs/sb311-ED-visit-costs-2021.pdf.

[21] U.S. Bureau of Labor Statistics CPI inflation calculator. https://www.bls.gov/data/inflation_calculator.htm.

[22] Centers for Medicare & Medicaid Services Hospital value-based purchasing program. https://www.cms.gov/medicare/quality/initiatives/hospital-quality-initiative/hospital-value-based-purchasing.

[23] Al-Sultani Z, Inglis TJ, McFadden B, Thomas E, Reynolds M (2025). Sepsis *in silico*: definition, development and application of an electronic phenotype for sepsis. J Med Microbiol.

[24] Kraus CK, Nguyen HB, Jacobsen RC, Ledeboer NA, May LS (2023). Rapid identification of sepsis in the emergency department. JACEP Open.

[25] Chen AY, Allison M, Puskarich M, Vilke GM, Taub P (2025). Risk factors associated with return sepsis admission following emergency department discharge with infection. Am J Emerg Med.

[26] Schneider J, Romanowsky J, Schuetz P, Stojanovic I, Cheng H (2020). Cost impact model of a novel multi-mRNA host response assay for diagnosis and risk assessment of acute respiratory tract infections and sepsis in the emergency department. J Health Econ Outcomes Revs.

[27] Davis RF, Navalkar KA, Poll T, Schultz MJ, Cremer OL Septicyte rapid in sepsis cases with malignancy or treated with antineoplastics / immunosuppressants. https://septicyte.com/wp-content/uploads/2021/01/IMM-009-SCCM-04-01-SC.pdf.

[28] Sato E, Fujimoto J, Tamaya T (2007). Expression of interferon-gamma-inducible protein 10 related to angiogenesis in uterine endometrial cancers. Oncology.

[29] Holoch PA, Griffith TS (2009). TNF-related apoptosis-inducing ligand (TRAIL): a new path to anti-cancer therapies. Eur J Pharmacol.

[30] Kobo O, Itzhaki I, Drescher MJ, Glazer J, Israeli A (2025). Non-clinical factors influencing discharge and admission decisions in the emergency department: a focus group study among Israeli physicians. BMC Emerg Med.

